# Lumped Element Model for Thermomagnetic Generators Based on Magnetic SMA Films

**DOI:** 10.3390/ma14051234

**Published:** 2021-03-05

**Authors:** Joel Joseph, Makoto Ohtsuka, Hiroyuki Miki, Manfred Kohl

**Affiliations:** 1 Institute of Microstructure Technology, Karlsruhe Institute of Technology (KIT), Postfach 3640, D-76021 Karlsruhe, Germany; joel.joseph@kit.edu; 2 Institute of Multidisciplinary Research for Advanced Materials, Tohoku University, Sendai 980-8577, Japan; makoto.ohtsuka.d7@tohoku.ac.jp; 3 Institute of Fluid Science, Tohoku University, Sendai 980-8577, Japan; hiroyuki.miki.c2@tohoku.ac.jp

**Keywords:** thermomagnetic energy generators, power generation, waste heat recovery, lumped-element modelling, magnetic shape memory films, Ni-Mn-Ga film, magnetization change, Curie temperature

## Abstract

This paper presents a lumped element model (LEM) to describe the coupled dynamic properties of thermomagnetic generators (TMGs) based on magnetic shape memory alloy (MSMA) films. The TMG generators make use of the concept of resonant self-actuation of a freely movable cantilever, caused by a large abrupt temperature-dependent change of magnetization and rapid heat transfer inherent to the MSMA films. The LEM is validated for the case of a Ni-Mn-Ga film with Curie temperature T_C_ of 375 K. For a heat source temperature of 443 K, the maximum power generated is 3.1 µW corresponding to a power density with respect to the active material’s volume of 80 mW/cm^3^. Corresponding LEM simulations allow for a detailed study of the time-resolved temperature change of the MSMA film, the change of magnetic field at the position of the film and of the corresponding film magnetization. Resonant self-actuation is observed at 114 Hz, while rapid temperature changes of about 10 K occur within 1 ms during mechanical contact between heat source and Ni-Mn-Ga film. The LEM is used to estimate the effect of decreasing T_C_ on the lower limit of heat source temperature in order to predict possible routes towards waste heat recovery near room temperature.

## 1. Introduction

With the introduction of the internet of things and wireless sensor networks, the world is moving towards an interconnected network of distributed devices that collect data of their environment and communicate via the internet. In most cases, autonomous operation is mandatory and power supply by batteries or cables is not desired [1,2]. Energy harvesting is considered a solution as it allows producing small amounts of power at site. Recovery of low-grade thermal energy is of special interest, which is mostly rejected as waste heat making up a huge portion of energy lost in the environment. Much of the unrecovered waste heat is in the low temperature regime (10–250 °C) [3,4,5]. Keeping a high conversion efficiency at small temperature difference is a major challenge, particularly when miniature dimensions are required. Thermoelectric harvesting technology is considered the state of the art in this realm, however, it suffers from scalability due to the need for large heat sinks that may exceed the size of the actual device by far [6,7,8]. 

Thermomagnetic energy generators (TMGs) have the potential to overcome the limitations of today’s miniaturized thermal energy harvesting systems. The device performance strongly depends on the magnetic material used for energy conversion that should exhibit, among others, a large change of magnetization Δ*M* at small temperature difference Δ*T* and a large thermal conductivity. A number of TMG devices make use of ferromagnetic materials, in particular gadolinium (Gd), showing a pronounced second-order ferromagnetic transition near the Curie point close to room temperature [9,10]. An interesting alternative are La-Fe-Si-based materials [11,12]. Recently, spin reorientation has been considered that exploits a temperature-induced change in the magnetic easy axis [13]. Magnetic shape memory alloys (MSMAs) are another promising material candidate that have been tailored to exhibit a large change of magnetization ΔM at small temperature difference ΔT [14,15,16,17]. One option is to make use of the steep increase in magnetization at the first-order transformation between non-ferromagnetic martensite and ferromagnetic austenite in metamagnetic alloys, which is highly attractive in the case of small hysteresis [18,19,20]. Another option is to utilize the large change of magnetization at the second-order transition, e.g., in the Heusler alloy Ni-Mn-Ga, which occurs without hysteresis [21,22].

Another critical aspect of device performance is the engineering of heat intake and dissipation for optimum energy conversion. In recent years several TMG concepts have been introduced that involve energy generation either indirectly via periodic mechanical motion such as rotation and oscillation or directly via thermal-to-magnetic energy conversion. Macroscale TMG demonstrator designs include thermomagnetic oscillators and linear harvesters showing oscillation cycles at a frequency of typically below 1 Hz [9,23]. Millimeter-scale TMG devices have the potential to operate at substantially higher frequencies due to the increased surface-to-volume ratio and, thus, allow for higher power output [10,19]. Recent demonstrators based on Ni-Mn-Ga films show promising results with magnetic power density close to 120 mW/cm^3^ with respect to the active layer volume [21]. The electrical power per footprint of the resonant TMG devices reached 50 µW/cm^2^ at a temperature change of only 3 K [22]. The large power density relies on rapid heat transfer of the film and the unique concept of resonant self-actuation of a film cantilever oscillating at high frequency in the order of 100 Hz. Keeping the condition of resonant self-actuation is a complex engineering task due to the strong coupling of mechanical, magnetic, thermal and electric performance, which demands for an appropriate model to describe the various interdependencies. 

In the following, we present a lumped element modeling (LEM) approach to investigate the interplay of the involved physical properties, in particular, the effects of heat intake and heat dissipation on the local temperature changes of the active material as well as the resulting changes of magnetization and force dynamics on power output. The main novelties of this investigation are describing the experimental performance characteristics of a TMG demonstrator at resonant self-actuation with high accuracy and predicting the effect of decreasing Curie temperature on the lower limit of heat source temperature. 

## 2. Material Properties and Operation Principle 

**Figure 1:**
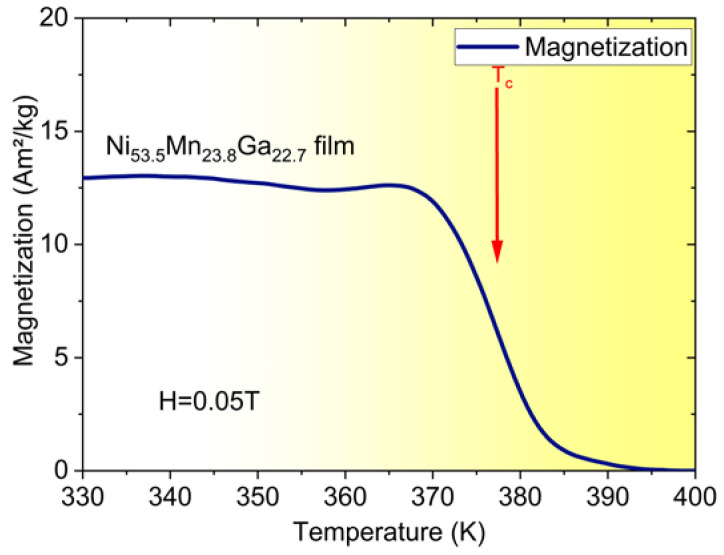
Magnetization versus temperature of a Ni-Mn-Ga film showing a sharp drop at the second-order ferromagnetic transition (Curie temperature T_C_) as indicated.

Ni–Mn–Ga films are chosen as a representative MSMA material. Ni–Mn–Ga films of 10 µm thickness are fabricated by RF magnetron sputtering in a high-purity argon atmosphere. The sputtering power is adjusted to control the Ni content and thus the phase transformation temperatures. Heat treatment conditions are tailored for optimal temperature-dependent change of magnetization ΔM/ΔT at the ferromagnetic transition. Details on film fabrication and film properties can be found in [24,25] and references therein. Figure 1 shows magnetization versus temperature of a Ni-Mn-Ga film at a low magnetic field of 0.05 T. The material is in martensitic and ferromagnetic state at room temperature. In the temperature range between 330 and 350 K, martensitic phase transformation occurs, whereby the reverse transformation from austenite to martensite at 350 K gives rise to the small step-like feature in Figure 1. At the Curie temperature T_C_ of about 375 K, magnetization shows a sharp change ΔM due to the ferromagnetic transition.

**Figure 2:**
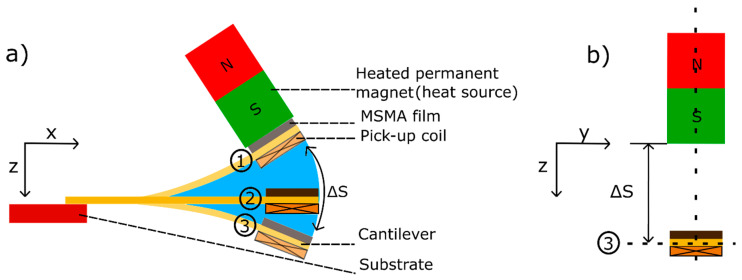
Layout and operation principle of the TMG device. A MSMA film of Ni-Mn-Ga and a pick-up coil are mounted at the cantilever front; (**a**) schematic side view of TMG device for three different deflection states (1)–(3), (**b**) front view for the maximum deflection state 3.

Figure 2 shows a schematic of the TMG device consisting of a cantilever beam attached to a substrate with both an active MSMA film and a pick-up coil attached to its freely movable end. A heated magnet is used to provide the magnetic force that attracts the free end of the cantilever towards the magnetic surface. Heat transfer occurs at mechanical contact causing a ferromagnetic transition in the MSMA film resulting in a reduction in magnetic force. In this case, the elastic force of the cantilever dominates and resets the cantilever. While oscillating back, the MSMA film cools and magnetic attraction force recovers. Consequently, a continuous oscillation occurs, which is sustained by the inertia force of the cantilever front. Thereby, the oscillatory motion supports cooling of the MSMA film. Resonant self-actuation sets in, when cooling and heating cause a sufficiently large change of magnetization and corresponding magnetic actuation force within a cycle. In this case, thermal energy is converted most efficiently into mechanical and magnetic energy, which in turn is converted into electrical energy by the pick-up coil.

One of the advantages of this TMG concept is that it is self-adapting to operating conditions like the source temperature and ambient temperature. When the source temperature increases, for instance, heat transfer increases causing an increase in oscillation amplitude and resonance frequency [22]. Yet, a major challenge is to maintain the condition of resonant self-actuation for optimum power output. In particular, any change of material parameters and dimensions may affect the resonance condition and, thus, may cause a deviation from optimum operation. Therefore, it is mandatory to understand the complex interplay of mechanical, thermal, and magnetic properties and their effect on the dynamics of heat transfer and cantilever motion. In the following, we discuss how this challenge can be met by lumped element modeling (LEM) of device performance. 

## 3. Lumped Element Model

The LEM has to describe the energy conversion processes in the TMG device by coupling mechanical motion of the cantilever front, magnetization changes of the MSMA film and heat flows during film heating and cooling. Thus, the model should allow for the optimization of the design parameters to obtain maximum power and efficiency by keeping the harvester operating at resonance. The LEM is implemented in SIMSCAPE (R-2018b, 2018, MATLAB, Karlsruhe, Germany) to calculate the mechanical, magnetic and thermal performances, which are described in the following. During simulation, the different physics sections interchange data for each position and time step. The modelling parameters are summarized in the Appendix A.

### 3.1. Mechanical Section

The mechanical deflection of the cantilever front is approximated as a one-dimensional motion of the center of mass at the movable cantilever front, whereby the lateral displacement due to bending is neglected. When operating the TMG device in vertical direction, the gravitational force acts on the movable mass, but it is negligible compared to magnetic and restoring forces. The net force $\;F_{net}\;$acting on the cantilever front is given by the magnetic force $F_{mag}$, inertia force, damping force and elastic force of the cantilever:

(1) $$F_{net}=m\frac{d^{2}x}{dt}+c\frac{dx}{dt}+kx+F_{mag}-F_{em}-F_{air}$$

Thereby, the movable mass *m* at the cantilever front is taken as [26]:

(2) $$m=\frac{33}{140}\times m_{cant}+m_{coil}+m_{film}$$

When the cantilever front gets in contact with the magnet surface, an additional elastic impact force occurs causing it to suddenly stop its movement at the surface. 

**Figure 3:**
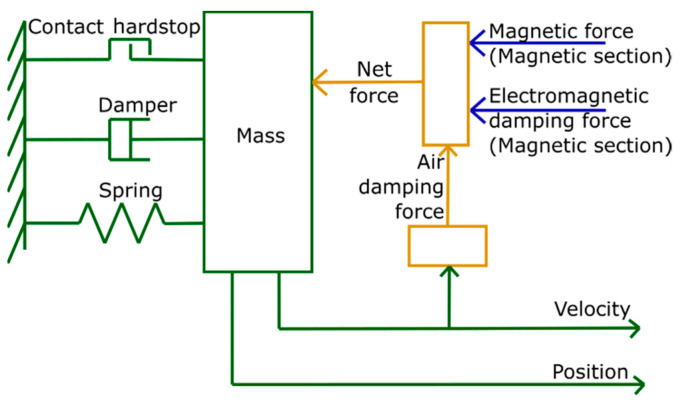
Mechanical section; the magnetic force and electromagnetic damping force from magnetic section are taken as input, while position and velocity are generated as output.

The mechanical section of the LEM is depicted schematically in Figure 3. The spring defines the stiffness of the beam cantilever *k* and the damper represents the structural damping with damping constant *c*. The impact force at contact is provided by the contact hard stop with contact stiffness *k_cont_* and impact damping constant *c_cont_*. Numerical values of these parameters used in the LEM simulation are summarized in Table A1. The beam is fixed at one end using a rigid reference. At the opposite freely movable end, the mass acts as a point load. The section takes the magnetic force and electromagnetic damping force as input parameters to compute the effective force at the cantilever front. Thereby, the damping forces caused by structural damping, viscous air damping $F_{air}$ [27] and electromagnetic damping $F_{em}$ [28] are taken into account. Based on the contributing forces, the equation of motion is solved to determine the position and velocity of the center of mass as the output parameters.

### 3.2. Magnetic Section

**Figure 4:**
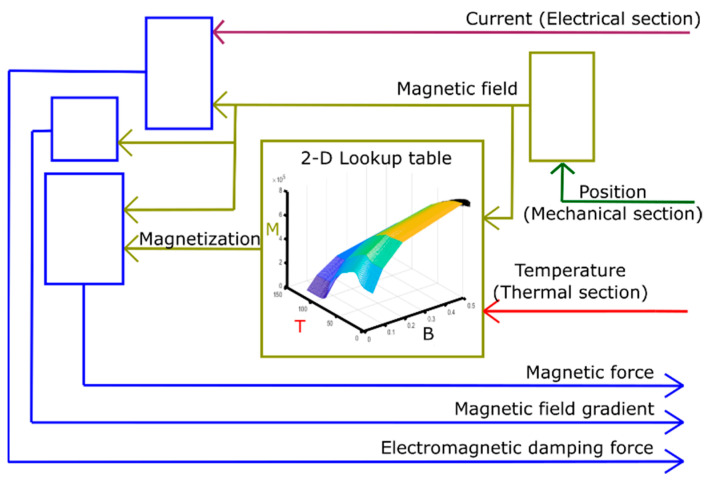
Magnetic section; temperature from thermal section, position from mechanical section and current from electrical section are taken in as inputs. The magnetic section computes magnetic force, magnetic field gradient and electromagnetic damping force as outputs. Magnetization data from material experiments are taken in as a lookup table.

Figure 4 shows a schematic of the magnetic section. Input parameters are the temperature of the MSMA film, the position of the cantilever front and the current of the pick-up coil. Experimental data on the temperature and magnetic field dependence of magnetization of the MSMA film are filed as a lookup table, which allows to select the most appropriate value of magnetization *M_T_* for the given input of magnetic field and temperature. *M_T_* enters into the calculation of the magnetic force $\;F_{mag}$ on the MSMA film. $F_{mag}$ is computed along the symmetry axis of the magnet in z direction [29]:

(3) $$F_{mag}=V_{mag}M_{T}\frac{\partial B_{z}}{\partial z},$$

whereby the cantilever deflection is assumed to be a one-dimensional motion. The course of magnetic field $B_{z}\;$and field gradient $\frac{\partial B_{z}}{\partial z}$ are analytically modeled as a function of position of MSMA film (distance from the magnet surface along z-direction) using equations given in [30]. $V_{mag}$ is the volume of the MSMA film. The electromagnetic damping is computed based on the current in the pick-up coil and the magnetic field. The output parameters are the magnetic force $F_{mag}$, which is a function of temperature and position of the MSMA film, and the electromagnetic damping force.

### 3.3. Thermal Section 

**Figure 5:**
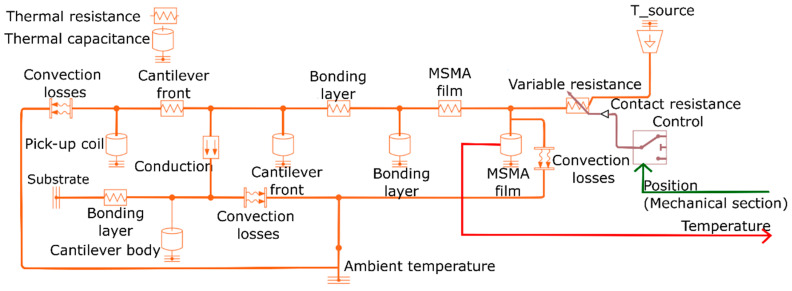
Thermal section; position of the MSMA film determined in the mechanical section is taken in as input and temperature is given out as output. Line connecting each thermal resistance and thermal capacitance represents bi-directional heat flow paths.

The thermal section of the LEM is used to study the heat flows in the TMG device. As shown in Figure 5, heat transfer in the system is modeled by representing the MSMA film, pick-up coil, bonding layers, cantilever front and body as thermal resistances and capacitances [31]. Heat transfer is indicated by bi-directional wire connections, whereby heat flows from higher to lower temperature. The intermittent heat transfer during mechanical contact between MSMA film and heat source is taken into account by control of a variable thermal resistance. For this purpose, a control box is connected to the heat source to model contact and non-contact instances during device operation based on the input of film position computed in the mechanical section. The thermal resistance strongly depends on the contact area and force. An appropriate value of thermal resistance is determined before by adapting simulated temperatures in test structures to corresponding experimental results. We thus obtain an empirical heat transfer coefficient *K_cond_* given in Table A1. The contact area is assumed identical to the film area for the case of a polished magnet surface due to the smooth surface finish of the MSMA film. Heat is dissipated from the MSMA film by heat convection to the surrounding air and by heat conduction to the cantilever. It turns out that the temperature of the cantilever front is close to the minimum temperature of the MSMA film during resonant self-actuation showing only a minor temperature change as will be discussed below.

The output of the thermal section is the time-dependent temperature of the MSMA film. It is important to note that the TMG device operates above the temperature of ferromagnetic transition of the MSMA film after reaching the condition of resonant self-actuation. In this case, the first order martensite to austenite transformation can be neglected. The thermal LEM section is important to understand the effects of MSMA material, geometry and other parameters on the heat flow characteristics. Optimum heat flow is crucial for resonant self-actuation and operation at optimum power output. 

### 3.4. Electrical Section 

**Figure 6:**
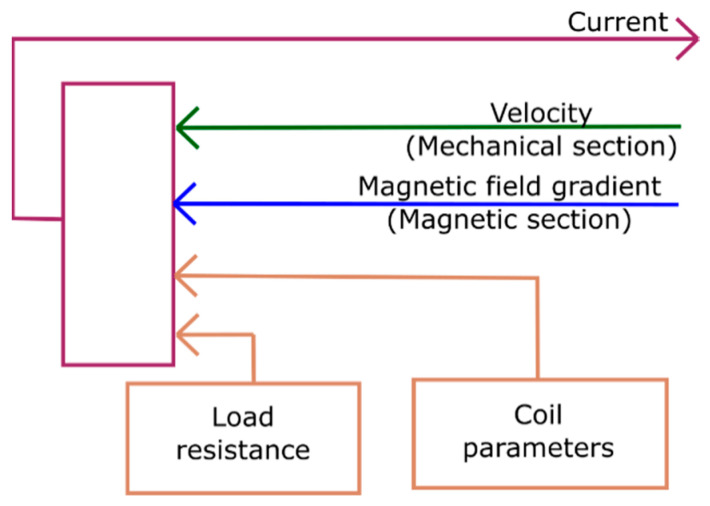
Electrical section; magnetic field gradient and velocity are taken in as inputs and a value for the current is generated as output. Coil parameters and load resistance are constants that are defined before.

Figure 6 shows a schematic of the electrical section. Based on the input of magnetic field gradient and velocity of the pick-up coil, it computes the induced voltage $V_{ind}$ using Faraday’s law [32]:

(4) $$V_{ind}=N_{coil}A_{coil}\frac{\partial B_{z}}{\partial z}\frac{\partial z}{\partial t},$$

whereby $N_{coil}$ and $A_{coil}$ denote the number of turns and area of the coil. The film velocity is denoted by $\frac{\partial z}{\partial t}$. The output of the electrical section is the induced current, which is determined from $V_{ind}$ and the internal coil resistance as a function of load resistance. The change in flux density due to the change in magnetization is neglected as it is a minor effect in the case of MSMA film. 

## 4. Performance Analysis

MSMA material and device dimensions are chosen to compare LEM results with experiments on a TMG demonstrator device in order to validate the LEM approach. The MSMA material under study is a Ni-Mn-Ga film having a thickness of 10 µm. The surface area of the film is 2 × 2 mm^2^ and of the cantilever is 2 × 5.7 mm^2^ as illustrated in Figure 2. The thicknesses of the cantilever and of the adhesive layer used to bond the film to the cantilever are 20 µm and about 15 µm, respectively. The cantilever is attached to a supporting ceramic substrate. For bonding, a non-conductive adhesive layer is used to increase heat transfer time and, thus, to retain most of the heat in the MSMA film that is transferred during mechanical contact to the heat source. The operation volume of the harvester is 17.18 mm^3^. The temperature of the heatable magnet (heat source temperature) is set to 443 K. The modeling parameters are summarized in Table A1 in the Appendix A. 

**Figure 7:**
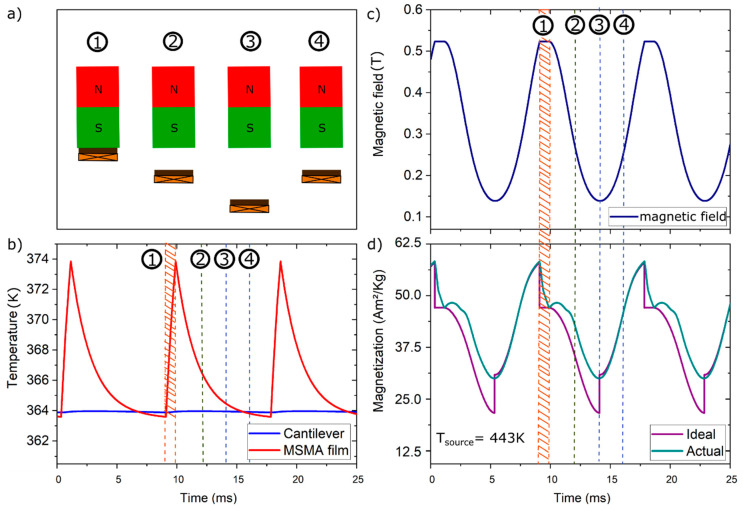
(**a**) Schematic illustration of cantilever motion to distinguish four stages (1)–(4) and time-resolved changes of (**b**) temperature in the Ni-Mn-Ga film and cantilever front, (**c**) magnetic field at the position of the Ni-Mn-Ga film and (**d**) magnetization of the Ni-Mn-Ga film.

In the following, the LEM is used to study the time-dependent changes of temperature, magnetic field and corresponding magnetization in the Ni-Mn-Ga film during thermomagnetic cycling. Four stages of cantilever motion may be distinguished as illustrated in Figure 7a. The first stage (1) corresponds to the instant of heat transfer, when the Ni-Mn-Ga film is in mechanical contact to the heat source. (2) Upon deflection from the magnet, the Ni-Mn-Ga film and pick-up coil are accelerated to maximum speed corresponding to a maximum induced voltage. The third stage (3) is reached at the maximum distance to the magnet. (4) A second maximum induced voltage occurs during attraction of the Ni-Mn-Ga film by the magnet, when the pick-up-coil is at maximum speed. 

The time-resolved change of temperature in the Ni-Mn-Ga film during resonant self-actuation is depicted in Figure 7b. During one oscillation cycle, the film shows a temperature change of about 10 K within a time duration of about 9 ms. While in mechanical contact (1), the temperature of the film exhibits a steep rise within about 1 ms. The subsequent temperature decrease occurs more slowly while passing through stages (2)–(4). The rather large temperature change is possible due to rapid heat transfer during mechanical contact to the heat source, while heat dissipation through heat conduction and convection is limited. The temperature of the cantilever front below the Ni-Mn-Ga film is close to the minimum temperature of the film and varies by less than 0.5 K. Most of the heat of the Ni-Mn-Ga film is dissipated by heat conduction through the cantilever and, therefore, the temperature of cantilever front represents the heat sink temperature of the TMG device. The temperature change in the pick-up coil is below 0.2 K per cycle.

Figure 7c,d show the corresponding changes of magnetic field and magnetization during thermomagnetic cycling. The time-dependent course of magnetic field reflects the deflection of the Ni-Mn-Ga film. While the film is in mechanical contact to the heat source (stage 1), the magnetic field is at its maximum (about 530 mT), and when the film reaches the maximum distance from the magnet of 1.8 mm, the magnetic field becomes minimal (about 130 mT). Fast-Fourier-Transform analysis of the simulated and experimentally measured deflection yields an oscillation frequency of 114 Hz. The time-dependent course of magnetization is more complex. Figure 7d shows the two cases of the actual cycle of the Ni-Mn-Ga film and the corresponding ideal cycle. In the ideal case of adiabatic conditions, heat exchange only occurs at mechanical contact to the heat source in stage (1) and in stage (3) assuming heat rejection to a heat sink. In this case, sharp changes of magnetization occur in stages (1) and (3) due to rapid temperature change. In stages (2) and (4), the magnetization follows the decrease and increase in magnetic field, respectively. In the actual cycle, a sharp drop in magnetization occurs in stage (1), while in stage (3) only a smooth increase in magnetization occurs. This behavior is due to the non-adiabatic operating conditions, as the film starts cooling as soon as it leaves the magnet surface and continues cooling during stages (2)–(4).

**Figure 8:**
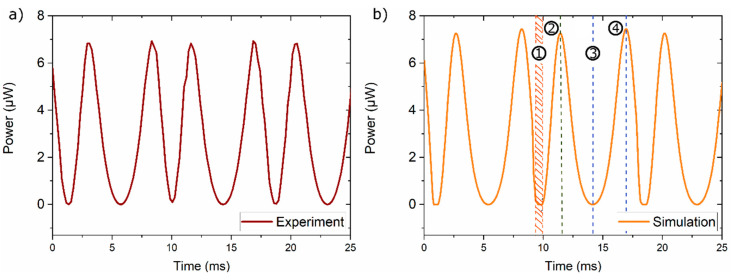
Experiment (**a**) and LEM simulation (**b**) of electrical power versus time of a TMG device based on a Ni-Mn-Ga film of 10 µm thickness. The four stages of cantilever motion (1)–(4) are highlighted (compare Figure 7a).

Figure 8a,b show the simulated and experimental electrical power of the TMG device versus time, respectively. Presented results are determined under stationary operation conditions, which is reached after a few minutes of continuous resonant oscillation and then persists for hours without noticeable change of performance. Two power maxima occur during one thermomagnetic cycle at the stages (2) and (4), where the Ni-Mn-Ga film deflects at maximum speed causing a direction-dependent positive and negative peak of alternating current, respectively. The first peak at stage (2) is slightly smaller compared to the peak at stage (4) due to the position-dependent difference in magnetic field. The maximum power generated is 3.1 µW corresponding to a power density with respect to the active material’s volume of 80 mW/cm^3^. The simulated course of electrical power agrees with the experiment with an accuracy better than 10 %, which validates the LEM approach. Additionally, time-dependent deflection, stroke and frequency (not shown here) show very good agreement. 

## 5. Discussion

The LEM approach allows to describe the coupled thermo-magneto-mechanical performance of the TMG device and to analyze each physical section. Here, we focus on the temperature change of the MSMA film, the time-dependence of magnetic field at the position of the film and on the corresponding film magnetization, which causes the change of magnetic actuation force required to maintain resonant self-actuation. The investigated TMG device is actuated by a Ni-Mn-Ga film of 10 µm thickness. In this case, time-dependent temperature characteristics reveal a rapid temperature increase of 10 K within about 1 ms, followed by a temperature decrease within about 8 ms. This performance results from the rapid heat intake during mechanical contact between magnet and Ni-Mn-Ga film, and the slower heat dissipation by convection and conduction via the bonding layer and cantilever. The much slower temperature decrease results from the non-conductive adhesive between cantilever front and Ni-Mn-Ga film, which acts as a thermal boundary layer and helps to retain most of the input heat. Optimum heat dissipation occurs when input and output heat flows are matching over time. One limitation of the model is that we have to assume the thermal contact resistance between magnet and film and also the convective heat transfer coefficient, as these parameters are hard to measure and difficult to deduce theoretically due to the number of unknown variables. Therefore, these parameters are adapted to the experiment, see Table A1 in the Appendix A. By this approach, realistic values are obtained that match with corresponding data in literature [33]. 

The heat intake depends on the impact force at contact to the magnet and, thus, on the actuation force, which is determined by the magnetic field gradient and magnetization. The maximum magnetization at contact with the magnet is 4.7 × 10^5^ A/m, which gives rise to a maximum attraction force of 5.48 mN. During contact, the magnetization rapidly drops by about 10^5^ A/m while the temperature increases adiabatically from about 364 K by about 10 K. The corresponding ratio ΔM/ΔT is about 10^4^ Am^−1^ K^−1^, which is consistent with the magnetization characteristic shown in Figure 1. It turns out that optimum operation occurs at the onset of ferromagnetic transition, where the magnetization is still high. Thus, sufficiently large magnetic forces are retained to complete the thermomagnetic cycle. The spring constant of the cantilever is adjusted to counterbalance the actuation and inertia forces, which gives rise to the rather high operation frequency of 114 Hz. 

**Figure 9:**
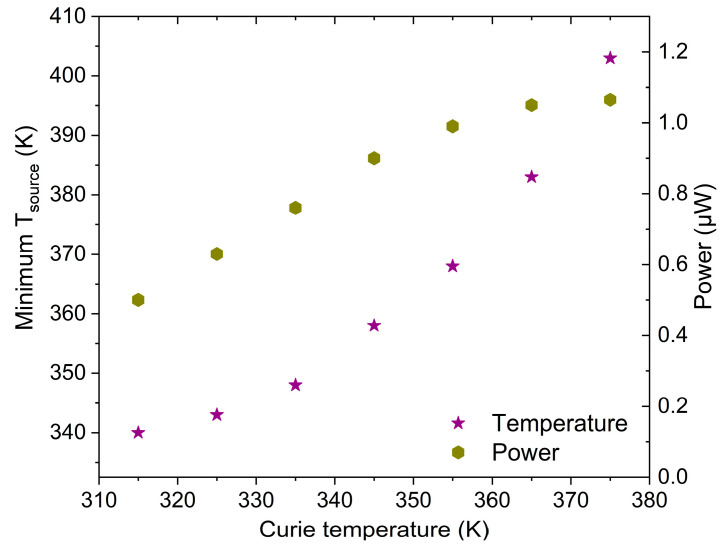
Minimum source temperature required for operation at various Curie temperatures.

The LEM simulation reveals an average electrical power output of 3.1 µW for a Ni-Mn-Ga film with Curie temperature T_C_ of 375 K and a heat source temperature of 443 K in agreement with the experiment. The material’s T_C_ sets the lower limit of heat source temperature T_source_ required to achieve resonant self-actuation. Previous investigations show that resonant self-actuation can be uphold for decreasing T_source_ down to about 400 K [17]. Power generation from waste heat at lower temperatures requires MSMA materials with lower T_C_. This gives rise to the interesting question, how the lower limit of T_source_ changes for decreasing T_C_. In order to address this question, the material’s T_C_ is decreased stepwise and the limit of T_source_ is evaluated in each case by LEM simulations without changing ΔM/ΔT and extrapolating heat transfer coefficients based on the dependence on T_source_ between 443 and 400 K. Under these assumptions, we obtain the characteristics of T_source_ limit and corresponding power output shown in Figure 9. When decreasing T_C_ down to 315 K, the LEM simulation predicts that resonant self-actuation can be uphold down to about 338 K, while the power output decreases to about 0.5 µW. This performance is mainly limited by the temperature-dependent magnetization change of the material ΔM/ΔT and heat transfer. 

## 6. Conclusions

We present a LEM approach to describe the coupled thermo-magneto-mechanical performance of a thermomagnetic generator (TMG) device that makes use of the large temperature-dependent change of magnetization at the ferromagnetic transition T_C_ of a MSMA film. The LEM is divided in sections to describe the various physical performances. The key requirement for maximum energy conversion and, thus, optimum power output is to meet the condition of resonant self-actuation, which is accurately described by the LEM. The LEM approach is validated by comparing LEM simulation results with experiments on a TMG demonstrator device using a Ni-Mn-Ga film of 10 µm thickness having a T_C_ of 375 K. For a heat source temperature of 443 K, the maximum power generated is 3.1 µW corresponding to a power density with respect to the active material’s volume of 80 mW/cm^3^. A time-dependent investigation of temperature of the MSMA film reveals rapid temperature changes of about 10 K caused by the heat flow during mechanical contact between magnet and Ni-Mn-Ga film and by matching of heat conduction via the bonding layer and cantilever. From the magnetization change Δ*M* and adiabatic temperature increase Δ*T* during contact the ratio ΔM/ΔT is determined to be about 10^4^ Am^−1^K^−1^. The simulated time dependence of magnetization indicates that optimum operation occurs at the onset of ferromagnetic transition, where the magnetization is high enough to generate sufficiently large magnetic forces to uphold resonant self-actuation. As the LEM is useful to analyze and optimize the device performance, it could be a useful tool to make performance predictions. Here, we estimate the effect of decreasing T_C_ on the lower limit of heat source temperature T_source_ and corresponding power output, which indicates that resonant self-actuation is possible down to source temperatures of about 338 K. Further optimization would require MSMA materials with enhanced thermomagnetic properties ΔM/ΔT and thermal interfaces with improved thermal contact to enable waste heat recovery near room temperature.

## Data Availability

Data sharing is not applicable to this article.

## Appendix A. Model Parameters

Table A1 summarizes all parameters used for LEM simulation. The parameters depend on the MSMA material and the detailed operation conditions of the TMG device. Table A1 also shows specific values used for LEM simulation of a TMG demonstrator device using a Ni-Mn-Ga film of 10 µm thickness with Curie temperature T_C_ of 375 K.

**Table A1:** Parameters used for LEM simulation.

Parameter	TMG Device Based on a Ni-Mn-Ga Film	Reference
Length of MSMA film	2 mm	This work
Width of MSMA film	2 mm	This work
Thickness of MSMA film	10 µm	This work
Density of MSMA material	8020 kg/m^3^	[34]
Length of the cantilever beam	5.7 mm	This work
Width of the cantilever beam	2 mm	This work
Thickness of the cantilever beam	20 µm	This work
Density of cantilever material	8500 kg/m^3^	[35]
Length of bonding layer	2 mm	This work
Width of bonding layer	2 mm	This work
Thickness of bonding layer	10 µm	This work
Density of bonding layer	1250 kg/m^3^	[36]
Young’s modulus of cantilever material (*E*)	1 × 10^11^ N/m^2^	[37]
Contact stiffness beam-magnet (*k_cont_*)	1 × 10^4^ N/m	This work
Structural damping (*c*)	1.12 × 10^−5^ Ns/m	This work
Impact damping (*c_cont_*)	0.1 Ns/m	This work
Thermal conductivity of MSMA material	23.2 W/mK	[38]
Specific heat capacity of MSMA material	490 J/kgK	[38]
Thermal conductivity of cantilever material	109 W/mK	[39]
Specific heat capacity of cantilever material	400 J/kgK	[35]
Thermal conductivity of bonding layer	0.33 W/mK	[36]
Specific heat capacity of bonding layer	2100 J/kgK	[36]
Area of thermal contact	4 mm^2^	This work
Max. Conductive heat transfer coefficient (*K_cond_*)	8400 W/m^2^K	This work
Convective heat transfer coefficient (*K_conv_*)	150 W/m^2^K	This work
Remanent magnetic field	1.07 T	This work
Number of turns of coil	400	This work
Area of coil	1.96 × 10^−6^ m^2^	This work
Electrical resistance of coil (internal resistance)	250 Ω	This work
Electrical load resistance	400 Ω	This work
Operation temperature	443 K	This work
